# Using meta-analysis and CNN-NLP to review and classify the medical literature for normal tissue complication probability in head and neck cancer

**DOI:** 10.1186/s13014-023-02381-7

**Published:** 2024-01-09

**Authors:** Tsair-Fwu Lee, Yang-Wei Hsieh, Pei-Ying Yang, Chi-Hung Tseng, Shen-Hao Lee, Jack Yang, Liyun Chang, Jia-Ming Wu, Chin-Dar Tseng, Pei-Ju Chao

**Affiliations:** 1 Medical Physics and Informatics Laboratory of Electronics Engineering, National Kaohsiung University of Science and Technology, No.415, Jiangong Rd., Sanmin Dist., Kaohsiung, 80778 Taiwan, ROC; 2 Graduate Institute of Clinical Medicine, Kaohsiung Medical University, Kaohsiung, 807 Taiwan, ROC; 3 Department of Medical Imaging and Radiological Sciences, Kaohsiung Medical University, Kaohsiung, 80708 Taiwan, ROC; 4 Department of Radiation Oncology, Linkou Chang Gung Memorial Hospital and Chang Gung University College of Medicine, Linkou, Taiwan, ROC; 5 Medical Physics at Monmouth Medical Center, Barnabas Health Care at Long Branch, Long Branch, NJ USA; 6 Department of Medical Imaging and Radiological Sciences, I-Shou University, Kaohsiung, 840 Taiwan, ROC; 7 Heavy Ion Center of Wuwei Cancer Hospital, Gansu Wuwei Academy of Medical Sciences, Gansu Wuwei Tumor Hospital, Wuwei, Gansu Province China; 8 Department of Medical Physics, Chengde Medical University, Chengde, Hebei Province China

**Keywords:** Meta-analysis, Natural language processing, Head and neck cancer, Squamous cell carcinoma of the head and neck, Normal tissue complication probability prediction, Convolutional neural networks, Artificial intelligence, Radiation therapy

## Abstract

**Purpose:**

The study aims to enhance the efficiency and accuracy of literature reviews on normal tissue complication probability (NTCP) in head and neck cancer patients using radiation therapy. It employs meta-analysis (MA) and natural language processing (NLP).

**Material and methods:**

The study consists of two parts. First, it employs MA to assess NTCP models for xerostomia, dysphagia, and mucositis after radiation therapy, using Python 3.10.5 for statistical analysis. Second, it integrates NLP with convolutional neural networks (CNN) to optimize literature search, reducing 3256 articles to 12. CNN settings include a batch size of 50, 50–200 epoch range and a 0.001 learning rate.

**Results:**

The study's CNN-NLP model achieved a notable accuracy of 0.94 after 200 epochs with Adamax optimization. MA showed an AUC of 0.67 for early-effect xerostomia and 0.74 for late-effect, indicating moderate to high predictive accuracy but with high variability across studies. Initial CNN accuracy of 66.70% improved to 94.87% post-tuning by optimizer and hyperparameters.

**Conclusion:**

The study successfully merges MA and NLP, confirming high predictive accuracy for specific model-feature combinations. It introduces a time-based metric, words per minute (WPM), for efficiency and highlights the utility of MA and NLP in clinical research.

**Supplementary Information:**

The online version contains supplementary material available at 10.1186/s13014-023-02381-7.

## Introduction

Advancements in radiation therapy techniques for head and neck cancer have significantly improved patients' quality of life [1]. However, potential complications such as dysphagia, xerostomia, and mucositis can hinder recovery and amplify adverse effects. Specifically, radiation-induced xerostomia substantially diminishes patients' well-being, leading to oral health issues and communication barriers [2].

To enhance the welfare of head and neck cancer patients, researchers are exploring innovative approaches, including artificial intelligence (AI) and predictive algorithms, to investigate potential risk factors for complications. This multidisciplinary research has proliferated a vast body of publications. For instance, a literature search using the terms "artificial intelligence" and "head and neck cancer" between 2013 and May 2022 yielded 734,207 related articles on WOS, indicating a marked upward trend.

Given the sheer volume of published literature, comprehensive understanding through traditional literature reviews becomes increasingly challenging. Therefore, systematic search and filtering methods are crucial. Optimized strategies involve meta-analysis (MA) for synthesizing literature information, quantitatively integrating high-quality data to create valuable annotated datasets, thereby providing robust quantitative evidence for clinical decision-making.

However, conducting an integrated MA is time-consuming and labor-intensive, particularly in literature screening [3]. Reviewers face the daunting task of sifting through a plethora of articles with varying degrees of expertise and clinical relevance. To enhance the efficiency and accuracy of MA, this study employs natural language processing (NLP) techniques. As a significant branch of Artificial Intelligence, NLP enables computers to understand human language and has proven its applicability across various domains [4]. Utilizing NLP can augment the quantitative capabilities of MA, minimize human errors, and automate the screening process. The primary aim of this approach is to improve analytical efficiency while reducing human error.

NLP accelerates literature reviews by adeptly categorizing pertinent articles. Numerous studies have improved machine learning methods using publicly accessible literature from 15 systematic reviews [5–8]. For instance, Yujia et al. employed various machine learning models to classify abstracts into two categories related to cancer risk in genetic mutation carriers (penetrance) or the prevalence of genetic mutations [3]. Impressively, they achieved over 88% accuracy in both models. Zhengyi et al. demonstrated that NLP-based methods could substantially reduce the review workload while maintaining the ability to identify relevant research [3]. However, to date, no NLP techniques have been specifically tailored for literature on complications following head and neck cancer radiation therapy or normal tissue complication probability (NTCP). Furthermore, there's a conspicuous lack of an annotated dataset for crafting a machine learning model dedicated to discerning relevant articles in this domain.

Our research aims to fill this gap by creating an annotated abstract dataset focusing on the likelihood of three common complications post-radiation therapy for head and neck cancer—mucositis, xerostomia, and dysphagia. We will employ machine learning-based NLP methods to classify abstracts into this annotated dataset. The ultimate goal is to minimize human error and enhance analytical efficiency.

## Materials and methods

### Research framework

**Fig. 1:**
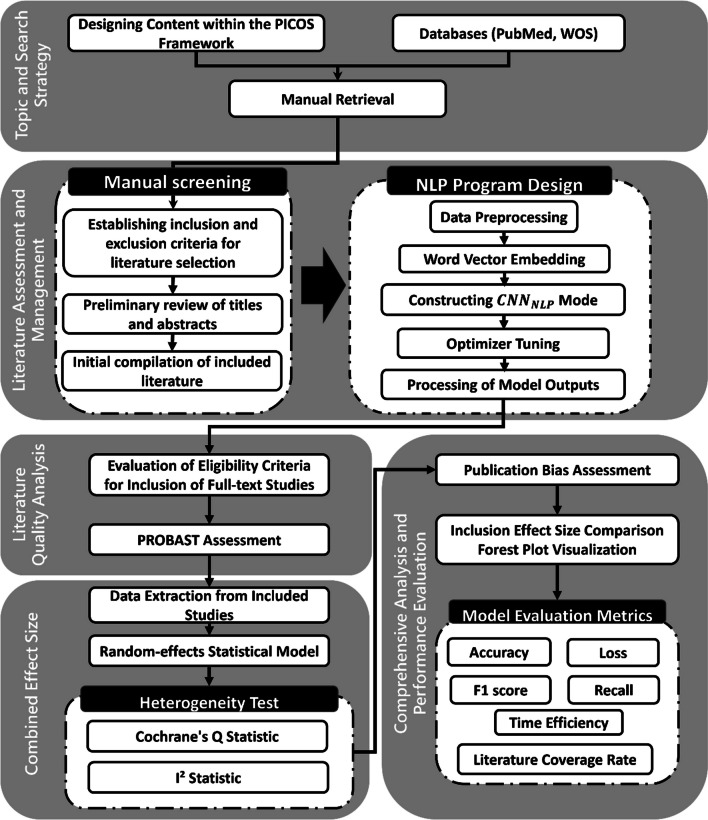
Research workflow diagram. *CNN* Convolutional neural networks, *NLP* Natural language processing, *WOS* Web of science, *PICOS* Patient characteristics, Intervention measures, Control group, Outcome

Our research process, based on MA, is divided into two parts, as depicted in Fig. 1. The first part employs MA to investigate NTCP predictive models for three common complications post-radiation therapy in head and neck cancer patients—xerostomia, dysphagia, and mucositis. The study encompasses patient demographics, methodologies, and outcomes, hypothesizing that significant variations may arise from different complication types, model choices, and predictive factors. By comparing various models and feature combinations, we aim to identify those with superior predictive capabilities, offering more effective predicting methods for clinical use. Statistical analyses are conducted using Python 3.10.5, with the null hypothesis stating that all model-feature combinations perform equally well in predicting complications, and the alternative hypothesis positing that at least one combination significantly outperforms the others.

The second part integrates natural language processing with convolutional neural networks (CNN) to enhance literature retrieval efficiency and result reliability. This approach aims to accelerate the time required for research on the NTCP of complications in head and neck cancer, offering quicker and more reliable insights for future studies and clinical applications.

### Eligibility criteria, information sources, and search strategy

**Fig. 2:**
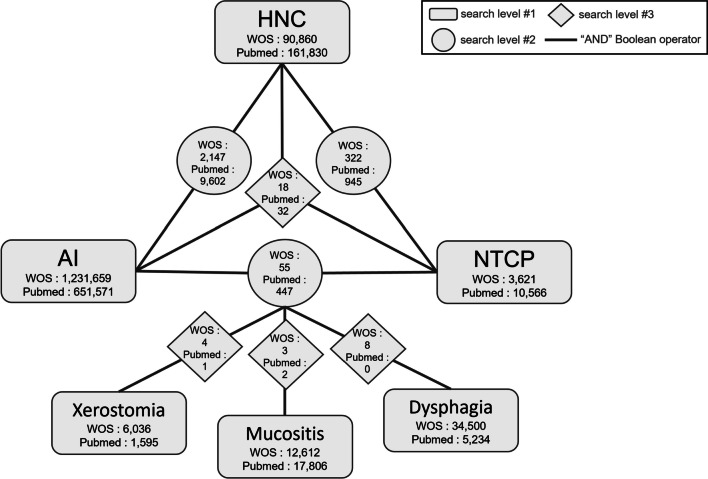
Search framework. *AI* Artificial intelligence, *HNC* Head and neck cancer, *NTCP* Normal tissue complication probability, *WOS* Web of science

This study outlines the research content on head and neck cancer patients using the PICOS framework [9] (patient characteristics, intervention measures, control group, outcome), as showed in Fig. 2. Patient characteristics focus on head and neck cancer patients; interventions encompass all radiation therapy techniques for treating this cancer; control groups are categorized into machine learning, deep learning model types, and feature factors; and the outcome metric targets the AUC of multivariate NTCP models. Given its non-RCT or CCT nature, the study falls under the category of prospective trials.

After formulating the research theme, database searches are conducted using relevant keywords, covering both titles and abstracts. Primary search keywords are organized into three layers: patient, method, and outcome, and are explored in conjunction with the PICOS framework. To ensure completeness, Boolean "AND" searches are specifically performed for combinations of complications with AI and NTCP. Beyond the PICOS framework, the study also employs PubMed's MeSH terms and related literature to broaden its scope. Boolean logic and faceted search techniques are used to break down the indexing problem into multiple thematic layers, establish inter-layer relationships, and employ Boolean "OR" for union operations, ensuring the comprehensiveness of the search results (detailed keywords are provided in Additional file 1: Table S1) [10–12].

### Selection process

Data extracted from each included study is determined through collaborative discussions among reviewers. One reviewer is responsible for data collection, while another performs cross-validation. The data encompasses authorship, publication year, types of complications, radiation therapy methods, employed models, features (prognostic factors), performance evaluation, as well as the study's contributions and conclusions.

### Data extraction and risk of bias (RoB) assessment

In our study, when evaluating the quality and potential biases of the literature for MA, we opted for the PROBAST tool (Prediction model Risk Of Bias ASsessment Tool) over the commonly used Cochrane risk of bias assessment (RoB) tool. This strategic choice was influenced by the realization that a significant portion of the studies-included did not align well with the criteria of the Cochrane tool due to their unique characteristics.

PROBAST evaluates four domains: participants, predictors, outcome, and analysis. The participants domain assesses the representativeness of the target population and selection bias; the predictors domain evaluates the selection, relevance, reliability, and handling of predictive factors; the outcome domain focuses on the measurement and definition of outcomes, assessing their accuracy and consistency; and the analysis domain reviews methods for model development and validation, including sample size, missing data handling, model calibration, and discriminative ability.

Bias risk assessment is conducted using the PROBAST Excel interface developed by Borja M. Fernandez-Felix [13], with risk determinations—low, high, or unclear—derived from responses to signaling questions. An overall low risk is assigned only if all domains are low-risk; a single high-risk domain results in an overall high risk; and an unclear risk in one domain with low risk in others leads to an overall unclear risk. If all model domains are low-risk but lack external validation, the risk is elevated to high; however, if based on extensive data with internal validation, it can be considered overall low-risk.

### Statistical methods

The MA in this study primarily contains the following key components:

I. Study Selection and Features: Provides an overview of the included sample size, time frame, model characteristics, and predictive factors used.
II. Combined Effect Size Results: Calculates the aggregated AUC, confidence intervals, and ANOVA p-values for the included studies, visually represented through forest plots to facilitate understanding of MA conclusions and statistical significance.
III. Heterogeneity Test Results: Utilizes Cochran's Q statistic [14] and I^2^ values [15] to assess study heterogeneity. A low p-value in the Q statistic indicates the presence of heterogeneity, while a higher I^2^ value quantifies greater inter-study variability.
IV. ANOVA analysis under random effects model: Calculates the effect size and variance for each study, using AUC as the benchmark. Determines the weight for each study, which is the reciprocal of the variance. Computes the overall effect size and variance. Calculates Q statistic, degrees of freedom, and I^2^ statistic. Conducts ANOVA analysis; if the Q statistic exceeds the degrees of freedom, significant inter-study differences exist, and F-values and p-values are calculated to assess the null hypothesis.
V. This process covers study selection, effect size aggregation, heterogeneity testing, and variance analysis under a random effects model, offering a comprehensive evaluation of the predictive models' ability to forecast the incidence of complications.

### Natural language processing (NLP) program design

To expedite the identification and retrieval of relevant literature while ensuring result reliability and accuracy, this study adopts a CNN for NLP, drawing inspiration from Yujia Bao's MA NLP model design [3]. This choice not only considers the nature of the data but also facilitates platform development, paving the way for the future integration of more deep learning models to enhance the classifier's accuracy and generalizability. In terms of abstract identification, the CNN model employed is capable of automatically learning language features from extensive text and achieving results across various tasks. Through word vector transformation and feature extraction, the CNN model effectively performs text classification and sentiment analysis. Key parameters used in this study include a batch size of 50, epoch range of 50–200, and a learning rate of 0.001.

### Data preprocessing

The data preprocessing in this study is divided into two main phases. First, abstracts and titles that have undergone manual retrieval and initial screening are allocated into training, validation, and test sets. The positive and negative samples in the training and validation sets are distributed at a 2:8 ratio, while the test set is further fine-tuned to a more realistic 15:85 ratio to better reflect the prevalence of irrelevant samples. Second, for word vector embedding, the text is converted into jsonl format and manually annotated and cleaned, including the removal of potentially misleading punctuation and special characters. These preprocessing steps optimize the text for word vector embedding input in the CNN model, facilitating subsequent NLP and analysis.

## Results

### Literature review and research selection

**Fig. 3:**
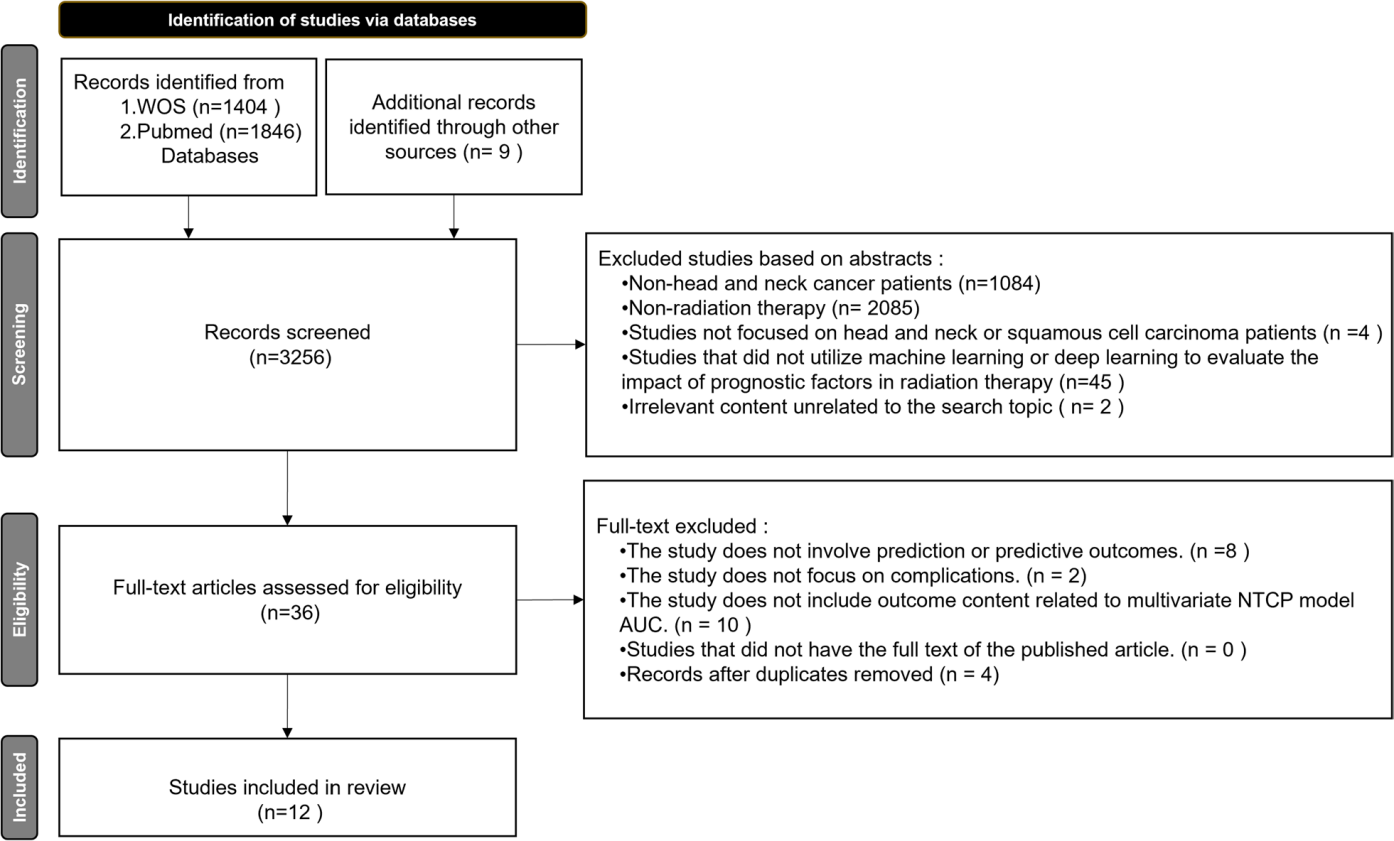
Article Selection flowchart. *WOS* Web of science

After searching the WOS and PubMed databases, this study initially identified 3,256 potentially relevant articles, as illustrated in Fig. 3. The first round of screening, based on titles, eliminated studies unrelated to head and neck cancer or radiation therapy, leaving 87 articles for the second round. The second round, focused on abstracts, further excluded studies not involving head and neck or squamous cell cancer patients, or those not utilizing machine learning or deep learning as evaluation tools, resulting in 36 articles for full-text review. During this phase, articles not addressing predictions, not focusing on complications, or lacking AUC-related outcomes for multivariate NTCP models were also excluded, along with duplicates. Ultimately, 12 articles were included for review [16–27].

### Performance of the CNN-NLP model

After comparing nine different optimizers, our study opted for Adamax (see Additional file 1: Table S2). With 50 epochs, Adamax achieved a Loss value of 0.51, an accuracy of 0.85, and an F1-Score of 0.75, along with a precision of 0.71. When the epochs were increased to 100, the accuracy and F1-Score improved to 0.87 and 0.79, respectively, while the precision reached 0.84. At 200 epochs, both accuracy and F1-Score peaked at approximately 0.94, clearly demonstrating the superior performance of the Adamax optimizer in the model.

After optimizer fine-tuning, as shown in Table 1, we evaluated coverage performance, which measures the overlap of identified studies under specific search subset conditions and assesses the efficacy of automated processing. We conducted tests on four different subsets, from WOS T1 to Pubmed T4, and compared the coverage rates when using Adam and Adamax optimizers across training cycles of 200, 100, and 50 epochs. In WOS T1, coverage was generally 0/9 regardless of the optimizer or training cycle, with Adam reaching a peak of 1/7 and low recognition frequency. In Pubmed T2, coverage was mostly 0/7, but a few articles were identified at epochs 100 and 50, not exceeding two in total. In WOS T3, Adam achieved a 3/4 coverage rate at 50 epochs, similar to Adamax. For Pubmed T4, Adam reached a 3/4 coverage rate at 100 epochs, while Adamax showed more stable performance across all training cycles, peaking at 2/4.

**Table 1:** Coverage results

Test set (Total samples)	optimizer	epoch	minimum computation time(s)	Highest coverage rate ( selected/total)	Identification Frequency (%)
WOS T1 ( 301)	Adam	200	343	1/9	20
		100	76.516		
		50	78.596		
	Adamax	200	351.103	0/9	0
		100	106.240		
		50	91.683		
Pubmed T2 ( 98)	Adam	200	337.565	1/7	40
		100	90.814		
		50	81.079		
	Adamax	200	331.719	2/7	40
		100	129.784		
		50	83.186		
WOS T3 ( 53)	Adam	200	351.416	3/4	80
		100	75.448		
		50	76.222		
	Adamax	200	345.064	2/4	80
		100	143.646		
		50	87.936		
Pubmed T4 ( 60)	Adam	200	334.702	3/4	40
		100	106.955		
		50	85.363		
	Adamax	200	336.015	2/4	80
		100	173.420		
		50	88.835		

*WOS* Web of science

In the aspect of words per minute (wpm) for literature review, our study introduces a more objective method for time quantification. Beyond providing a standardized metric for future research, we also employ unit conversion and a deep learning-based Natural Language Text Classifier for temporal comparisons. In Table 2, we also calculated and compared the time spent on alternative tasks, converting wpm results to seconds, the details for the screening speed measured in WPM can be seen in Additional file 1: Table S3. We then contrasted this with the average time needed for text recognition during preprocessing in T1-T4 test sets using an Adamax-optimized CNN-NLP model. As shown in Table 1, despite considerations like text recognition capabilities, the time efficiency gained through NLP shows a significant, intuitive difference. (Code for WPM Calculation Algorithm captured from the monitor is shown in Additional file 1: Figure S1).

**Table 2:** Time difference comparison between manual and nlp classifier approaches

Test Set ID	Data source	Number of entries	Word count covered	Manual time spent (seconds)	Average time spent by CNN-NLP (seconds)	CNN-NLP Relative to manual time spent Ratio
T1	WOS	301	88,861	48,376	164	1:294
T2	Pubmed	98	36,991	20,102	160	1:126
T3	WOS	53	13,510	7,349	167	1:44
T4	Pubmed	60	22,804	12,404	172	1:72

*CNN* Convolutional neural networks, *NLP* Natural language processing, *WOS* Web of science

### Features and model methods: systematic review

As shown in Table 3, the "studies-included" feature table aligns with the three dimensions of the MA issue discussed in our Materials and Methods section. In addition to the authors and publication years, the table also encompasses demographic characteristics, complications, types of radiation therapy techniques, algorithmic combinations in predictive models, predictive performance, and selected predictive factors. The systematic review ultimately included a total of 12 studies [16–27].

**Table 3:** Features for the included studies

Author (Year)	complications	Sample size	treatment	model/ algorithm	AUC(CI)	Prognostic factors & Feature Variables	Significant contributions and findings
Hubert S. Gabryś et al. [16]	Xerostomia	153	IMRT	LR-L1 LR-L2 LR-EN kNN SVM ET GTB	*Early Stage (0–6 months):* LR-L1 AUC Validation: 0.56 LR-L2 AUC Validation: 0.46 LR-EN AUC Validation: 0.54 kNN AUC Validation: 0.65 SVM AUC Validation: 0.57 ET AUC Validation: 0.44 GTB AUC Validation: 0.55 *Late Stage (6–15 months):* LR-L1 AUC Validation: 0.63 LR-L2 AUC Validation: 0.60 LR-EN AUC Validation: 0.56 kNN AUC Validation: 0.62 SVM AUC Validation: 0.52 ET AUC Validation: 0.55 GTB AUC Validation: 0.65 *Long-term (15-24 months):* LR-L1 AUC Validation: 0.86 LR-L2 AUC Validation: 0.86 LR-EN AUC Validation: 0.83 kNN AUC Validation: 0.74 SVM AUC Validation: 0.79 ET AUC Validation: 0.88 GTB AUC Validation: 0.77 *Longitudinal Long-term (15–24 *months*):* LR-L1 AUC Validation: 0.52 LR-L2 AUC Validation: 0.39 LR-EN AUC Validation: 0.52 kNN AUC Validation: 0.58 SVM AUC Validation: 0.57 ET AUC Validation: 0.51 GTB AUC Validation: 0.63	*Demographics:* Age, Gender, Salivary Gland Shape, Volume, Sphericity, Eccentricity *Volume Dose Histogram:* Mean, Distribution, Skewness *Spatial Dose Gradient:* Gradient x, Gradient y, Gradient z Spatial Dose *Distribution:* η200, η020, η002 Spatial Dose *Correlation:* η110, η101, η011 Spatial Dose *Skewness:* η300, η030, η003 *Spatial Dose Co-skewness:* η012, η021, η120, η102, η210, η201	1. The integration of organ and dose shape descriptors has a positive impact on predicting xerostomia 2. The prediction of xerostomia is dependent on patient-specific and non-dosimetric factors, emphasizing the importance of personalized data for risk assessment 3. These insights offer detailed machine learning methodologies that are valuable for future radiomics and dosiomics in the establishment of NTCP (Normal Tissue Complication Probability) models
Tsair-Fwu Lee et al. ( 2014)	Xerostomia	206	IMRT	LASSO & Logistic Regression	*XER3m (LASSO-Suboptimal) Model:* Number of factors is 3 AUC is 0.84 *XER3m (LASSO-Optimal) Model:* Number of factors is 8 AUC is 0.86 *XER3m (Likelihood) Model:* Number of factors is 9 AUC is 0.85 *XER12m (LASSO-Suboptimal) Model:* Number of factors is 5 AUC is 0.84 *XER12m (LASSO-Optimal) Model:* Number of factors is 9 AUC is 0.87 *XER12m (Likelihood) Model:* Number of factors is 11 AUC is 0.86	*XER3m Related Factors:* Dmean-c, Dmean-i, Age, Economic Status, T Stage, AJCC Stage, Smoking, Education Level, Chemotherapy (C/T), Node Classification, Baseline Xerostomia, SIB or SQM, Gender, Family History, Marital Status *XER12m Related Factors:* Dmean-i, Dmean-c, Smoking, T Stage, Baseline Xerostomia, Alcohol Issues, Family History, Node Classification, Gender, Age, Economic Status, Chemotherapy (C/T), AJCC Stage, Marital Status, SIB or SQM	1. Utilizing the Least Absolute Shrinkage and Selection Operator (LASSO) to construct a multivariate logistic regression model effectively predicts the incidence of moderate to severe xerostomia in head and neck cancer patients undergoing Intensity-Modulated Radiation Therapy (IMRT) 2. Through LASSO, eight prognostic factors were identified for the 3-month time point: Dmean-c, Dmean-i, age, financial status, T-stage, AJCC stage, smoking, and education. For the 12-month time point, nine prognostic factors were identified: Dmean-i, education, Dmean-c, smoking, T-stage, baseline xerostomia, alcohol consumption, family medical history, and lymph node classification 3. During the process of selecting the optimal number of prognostic factors via LASSO, fine-tuning was performed using the Hosmer–Lemeshow test and AUC. For the 3-month time point, three optimal prognostic factors were selected: Dmean-c, Dmean-i, and age. For the 12-month time point, five optimal prognostic factors were selected: Dmean-i, education, Dmean-c, smoking, and T-stage 4. The overall performance of the NTCP model at both time points, as indicated by scaled Brier scores, Omnibus, and Nagelkerke R2 metrics, met certain standards and aligned with expected values 5. The multivariate NTCP model using LASSO was confirmed to be effective for predicting xerostomia in patients evaluated post-IMRT
Tsair-Fwu Lee et al. ( 2014)	Xerostomia	152 (HNSCC) 84 (NPC)	3D-CRT IMRT	LASSO & Logistic Regression	*XER HNSCC-3 m Model:* Number of Factors = 3 AUC = 0.88 (Range: 0.86–0.91) *XER HNSCC-12 m Model:* Number of Factors = 3 AUC = 0.98 (Range: 0.97–0.98) *XER NPC-3 m Model:* Number of Factors = 4 AUC = 0.87 (Range: 0.83–0.90) *XER NPC-12 m Model:* Number of Factors = 3 AUC = 0.96 (Range: 0.95–0.97)	Dmean-c Dmean-i Age Economic Status T-Stage Education Level	The multivariate Normal Tissue Complication Probability (NTCP) model developed using the Least Absolute Shrinkage and Selection Operator (LASSO) effectively predicts the incidence of moderate to severe xerostomia in patients with Head and Neck Squamous Cell Carcinoma (HNSCC) and Nasopharyngeal Carcinoma (NPC) undergoing Intensity-Modulated Radiation Therapy (IMRT) Through LASSO, higher AUC performance was retained while selecting the fewest predictive factors, resulting in the establishment of four predictive models In all models, the average dose to the contralateral and ipsilateral salivary glands was chosen as the most important predictive factor. Other selected clinical and socio-economic factors include age, financial status, T-stage, and educational level The multivariate logistic regression model using LASSO techniques can improve the prediction of the incidence of xerostomia in HNSCC and NPC patients The predictive model developed for HNSCC cannot be directly applied to the NPC population undergoing IMRT and vice versa, necessitating validation
Lisanne V. van Dijk et al. ( 2016)	Xerostomia	249	3D-CRT IMRT VMAT	LASSO & Logistic Regression	*XER12m Model without IBM Discrimination:* AUC = 0.75 ( 0.69–0.81) *XER12m Model with IBM Discrimination:* AUC = 0.77 ( 0.71–0.82) *XER12m Model without IBM Validation:* AUC boot = 0.74 *XER12m Model with IBM Validation:* AUC boot = 0.76	*CT Image Biomarkers (IBMs)* *Short Run Emphasis (SRE):* An image biomarker (IBM) that measures the heterogeneity of the parotid gland tissue Additional Parameters: *Mean Contra-lateral Parotid Gland Dose:* The average radiation dose received by the contra-lateral parotid gland during treatment *Maximum CT Intensity of the Submandibular Gland:* The highest computed tomography (CT) intensity value recorded for the submandibular gland *Mean Dose to Submandibular Glands:* The average radiation dose received by the submandibular glands during treatment	Existing models for predicting late-stage patient assessment of moderate to severe xerostomia (XER12m) and oral mucosal hypersecretion (STIC12m) after radiation therapy are primarily based on dose-volume parameters and baseline xerostomia (XERbase) or oral mucosal hypersecretion (STICbase) scores. However, the aim of the study is to improve these predictions by using patient-specific features based on CT image biomarkers (IBM) The research team prospectively collected planning CT scans and patient assessment outcome measurements for 249 head and neck cancer patients undergoing definitive radiation therapy (with or without systemic therapy) These potential image biomarkers (IBM) represent the geometric features, CT intensity, and textural characteristics of the salivary glands and submandibular glands Lasso regularization was used to create multivariate logistic regression models, and internal validation was performed through bootstrapping By adding the image biomarker "Short Run Emphasis" (SRE), which quantifies the heterogeneity of salivary gland tissue, to the average contralateral salivary gland dose and baseline xerostomia model, significant improvements were made in predicting xerostomia at 12 months For predicting oral mucosal hypersecretion at 12 months, researchers selected the maximum CT intensity of the submandibular gland as another image biomarker, in addition to baseline hypersecretion and the average dose to the submandibular gland By introducing image biomarkers representing the heterogeneity and density of the salivary glands, researchers improved predictions for xerostomia and oral mucosal hypersecretion at 12 months Providing image biomarkers can further guide the patient-specific response of healthy tissue to radiation doses in research
Stefano Ursino et al ( 2021)	Dysphagia	38	RT IMRT	LRC SVC RFC	*Predicting Dysphagia at 6 months:* SVC: AUC = 0.82 LRC: AUC = 0.80 RFC: AUC = 0.83 *Predicting Dysphagia at 12 months:* SVC: AUC = 0.85 LRC: AUC = 0.82 RFC: AUC = 0.94	Dose-Volume Histogram (DVH) features of the throat (SWOARs) Dose of Swallowing Risk Organs (SWOARs) Baseline and Post-Radiation 6 and 12 Months Penetration-Aspiration Score (P/A-VF)	Researchers developed a predictive model for Radiation-Induced Dysphagia (RID) based on Videofluoroscopy (VF) by incorporating Dose-Volume Histogram (DVH) parameters of Swallowing Risk Organs at Risk (SWOARs) into machine learning analysis The RID predictive model was developed using the dose of nine swallowing risk organs and the Penetration-Aspiration Score (P/A) from VF data at 6 and 12 months post-treatment Seventy-two dose features were extracted for each patient from the DVH and were analyzed using Linear Support Vector Classification (SVC), Logistic Regression Classification (LRC), and Random Forest Classification (RFC) Among 38 patients, the DVH features of SWOARs showed relevance at both 6 months (SVC's AUC 0.82; LRC's AUC 0.80; RFC's AUC 0.83) and 12 months (SVC's AUC 0.85; LRC's AUC 0.82; RFC's AUC 0.94) At 6 months, the SWOARs with the highest relevance and their corresponding features included the base of the tongue (V65 and Dmean), superior and middle constrictor muscles (V45, V55, V65, Dmp, Dmean, Dmax, and Dmin), and salivary glands (Dmean and Dmp). At 12 months, the features with the highest relevance included middle and inferior constrictor muscles (V55, Dmin, and Dmean; and V55, V65, Dmin, and Dmax), glottis (V55 and Dmax), laryngeal muscles (Dmax), and cervical esophagus (Dmax) A RID predictive model was trained and cross-validated, demonstrating high discriminative ability at both 6 and 12 months post-radiation therapy
Jamie A. Dean et al. ( 2018)	Dysphagia	263	3D-CRT IMRT	PLR SVC RFC	*6 months following RT:* PLRstandard: AUC = 0.82 ± 0.04 SVCstandard: AUC = 0.82 ± 0.04 RFCstandard: AUC = 0.78 ± 0.05 PLRspatial: AUC = 0.75 ± 0.08 SVCspatial: AUC = 0.74 ± 0.08 RFCspatial: AUC = 0.75 ± 0.05	PM receiving > 1 Gy/fraction	Researchers have proposed a model capable of predicting the severity of acute dysphagia in individual patients, which can be used to guide clinical decisions The goal of the study is to establish a model incorporating spatial dose metrics that can offer guidelines for radiation therapy planning, aiming to reduce the incidence of severe swallowing difficulties The researchers used radiation therapy doses to the pharyngeal mucosa (PM), including dose-volume and spatial dose metrics, along with clinical data, to develop a model for severe acute dysphagia Penalized Logistic Regression (PLR), Support Vector Classification (SVC), and Random Forest Classification (RFC) models were generated and internally (173 patients) and externally (90 patients) validated It was determined that the volume of the pharyngeal mucosa receiving moderate and high doses (greater than 1 Gy/fraction) is most correlated with severe acute dysphagia. In radiation therapy planning, these volumes should be minimized as much as possible to reduce the occurrence of severe acute dysphagia The performance of the Penalized Logistic Regression model using dose-volume metrics (PLR_standard) was comparable to more complex models and demonstrated excellent discriminative ability in external validation (Area Under the Curve, AUC = 0.82)
Jamie A. Dean et al. ( 2016)	Mucositis	351	RT (Not Specifically Stated)	PLR SVC RFC	PLRstandard: AUC = 0.72 ± 0.09 SVCstandard: AUC = 0.72 ± 0.09 RFCstandard: AUC = 0.71 ± 0.09 PLRspatial: AUC = 0.72 ± 0.09 SVCspatial: AUC = 0.71 ± 0.09 RFCspatial: AUC = 0.70 ± 0.09	Volumes of oral cavity receiving intermed—high dose	The aim of this study is to generate a predictive model for severe acute oral mucositis using spatial dose metrics and machine learning, which can guide clinical decision-making and inform treatment planning Researchers used radiation therapy dosages (dose-volume and spatial dose metrics) and clinical data to generate predictive models. They compared the performance of penalized logistic regression, support vector classification, and random forest classification models The performance of the standard dose-volume-based model was not significantly different from models that included spatial information. The discriminative ability was similar across all models, but the standard random forest classification model had the best calibration The average AUC and calibration slope for this model were 0.71 (SD = 0.09) and 3.9 (SD = 2.2), respectively The volume of the oral cavity receiving moderate and high doses is correlated with severe oral mucositis Reducing the volume of the oral cavity receiving moderate and high doses may potentially reduce the incidence of oral mucositis
Ivo Beetz et al. (2012)	Xerostomia	178	IMRT	M-LR	XER6m Model AUC = 0.68 (0.60–0.76)	Moderate to severe dry mouth (XER M6) and sticky saliva (STIC M6) were assessed at 6 months before and after treatment using the EORTC QLQ-H&N35 questionnaire (For all questions, including those related to dry mouth and sticky saliva, a 4-point Likert scale was used.) The main predictive factors for dry mouth are the average dose to the contralateral salivary gland and baseline dry mouth The main predictive factors for sticky saliva are the average dose to the contralateral submandibular gland, the sublingual gland, and the minor salivary glands of the soft palate	This is a multi-center prospective study aimed at developing a multivariate logistic regression model The purpose of the study is to predict the risk of xerostomia and sticky saliva in patients with head and neck cancer 6 months after receiving IMRT. The study covers 178 patients with head and neck cancer. The results show that 51.6% of patients experienced xerostomia after treatment; 35.6% of patients reported issues with sticky saliva The main predictive factors for xerostomia are the average dose to the contralateral salivary gland and baseline xerostomia The main predictive factors for sticky saliva are the average dose to the contralateral submandibular gland, sublingual gland, and minor salivary glands in the soft palate The model proposed in this study can serve as a reference for optimizing future IMRT treatments Moderate to severe xerostomia (XER M6) and sticky saliva (STIC M6) were assessed using the EORTC QLQ-H&N35 questionnaire before and 6 months after treatment For all questions, including those related to xerostomia and sticky saliva, a 4-point Likert scale was used
Ivo Beetz et al. [24]	Xerostomia	165	IMRT 3D-CRT	M-LR	XER6m Model AUC = 0.82 (0.76–0.89)	Moderate to severe dry mouth (XER M6) and sticky saliva (STIC M6) were assessed at 6 months before and after treatment using the EORTC QLQ-H&N35 questionnaire (For all questions, including those related to dry mouth and sticky saliva, a 4-point Likert scale was used.)	Dose distributions in minor salivary glands during 3D-CRT have limited impact on patient-rated salivary dysfunction symptoms Beyond the parotid and submandibular glands, only the sublingual glands showed a significant association with sticky saliva Reliable risk estimation needs other factors like age and baseline subjective scores Including these selected factors in predictive models enhances model performance significantly over just using dose volume histogram parameters
Kuo Men et al. [19]	Xerostomia	784	IMRT	3D rCNN	XER12m Model: AUC = 0.84 (0.74–0.91) No contour—AUC = 0.82 (0.72–0.90) No CT- AUC = 0.78 (0.67–0.88)	A subset of 40 images from the RTOG 0522 clinical trial had their features automatically extracted through deep learning	A toxicity prediction model using 3D rCNN was developed and evaluated The model extracted low- and high-level spatial features from CT planning images, radiation therapy dose distributions, and contours with 3D filters The proposed model showed promising results in predicting xerostomia Future studies focusing on more accurate definitions of xerostomia-associated regions can enhance the model's performance
Benjamin S Rosen et al. [26]	Xerostomia	105	VMAT	PLR	*Prediction of XER12m for* ≥ *1 grade xerostomia using Dose/Clinical model (DVH/Clinical):* AUC = 0.709 (95% CI, 0.603–0.815) *Prediction of XER12m with added Radiomics model (DVH/Clinical* + *Radiomics):* AUC = 0.719 (95% CI, 0.603–0.830) *Prediction of XER12m for* ≥ *2 grade xerostomia using Dose/Clinical model (DVH/Clinical):* AUC = 0.692 (95% CI, 0.615–0.770) *Prediction of XER12m with added contralateral salivary gland changes slightly improved predictive performance (DVH/Clinical* + *Radiomics):* AUC = 0.776 (95% CI, 0.643–0.912)	CBCT Image Features Patient Demographics Follow-up and Clinical Outcomes	1. A methodology has been introduced for using on-board CBCT to measure treatment-related PG changes during HNC radiotherapy 2. Early treatment CBCT measurements of PG density changes were linked to long-term xerostomia 3. These CBCT-measured changes offer better predictions than PG dose alone 4. The CBCT analysis can be conducted with minimal additional cost, making it a viable option for an adaptive radiotherapy platform
Khadija Sheikh et al [27]	Xerostomia	266	IMRT VMAT TomoTherapy	LASSO + Generalized linear models (multiple LR)	XER3m: DVH-AUC = 0.63 (0.51–0.81) CT-AUC = 0.57 (0.45–0.71) MR-AUC = 0.66 (0.54–0.82) CT + MR-AUC = 0.70 (0.57–0.82) DVH + CT-AUC = 0.56 (0.40–0.68) DVH + CT + MR-AUC = 0.60 (0.50–0.73) Clinical + CT + MR-AUC = 0.73 (0.62–0.86) Clinical + DVH + CT + MR-AUC = 0.68 (0.52–0.80)	IBMs (Image Biomarkers) CT and MR Imaging Dose-Volume Histogram (DVH) Parameters	1. Baseline image features from both parotid and submandibular glands can potentially serve as clinical surrogates for baseline function 2. Features from the submandibular glands might offer insights into unstimulated salivary function, enhancing predictions of post-RT xerostomia susceptibility 3. While combining all data showed a trend towards better prediction, further research is needed to ascertain the advantages of merging imaging modalities for xerostomia prediction 4. Prediction models based on these features can deepen our comprehension of radiation-induced xerostomia and aid in tailoring radiation treatment plans to reduce toxicity

*XER3m* Xerostomia around the 3-month time point, *XER6m* Xerostomia around the 6-month time point, *XER12m* Xerostomia around the 12-month time point, *Dmean-i* Average dose to the ipsilateral parotid gland, *Dmean-c* Average dose to the contralateral parotid gland, *LR-L1* Logistic regression with L1 penalty, *LR-L2* Logistic regression with L2 penalty, *LR-EN* Logistic regression with elastic net penalty, *kNN* k-Nearest neighbors, *SVM* Support vector machine, *ET* Extra-trees, *GTB* Gradient tree boosting, *LRC* Logistic regression classification, *SVC* Support vector classification, *RFC* Random forest classification, *M-LR* Multivariate logistic regression, *3D rCNN* 3-dimensional residual convolutional neural network, *LR* Logistic regression, *MR* Magnetic resonance

**Fig. 4:**
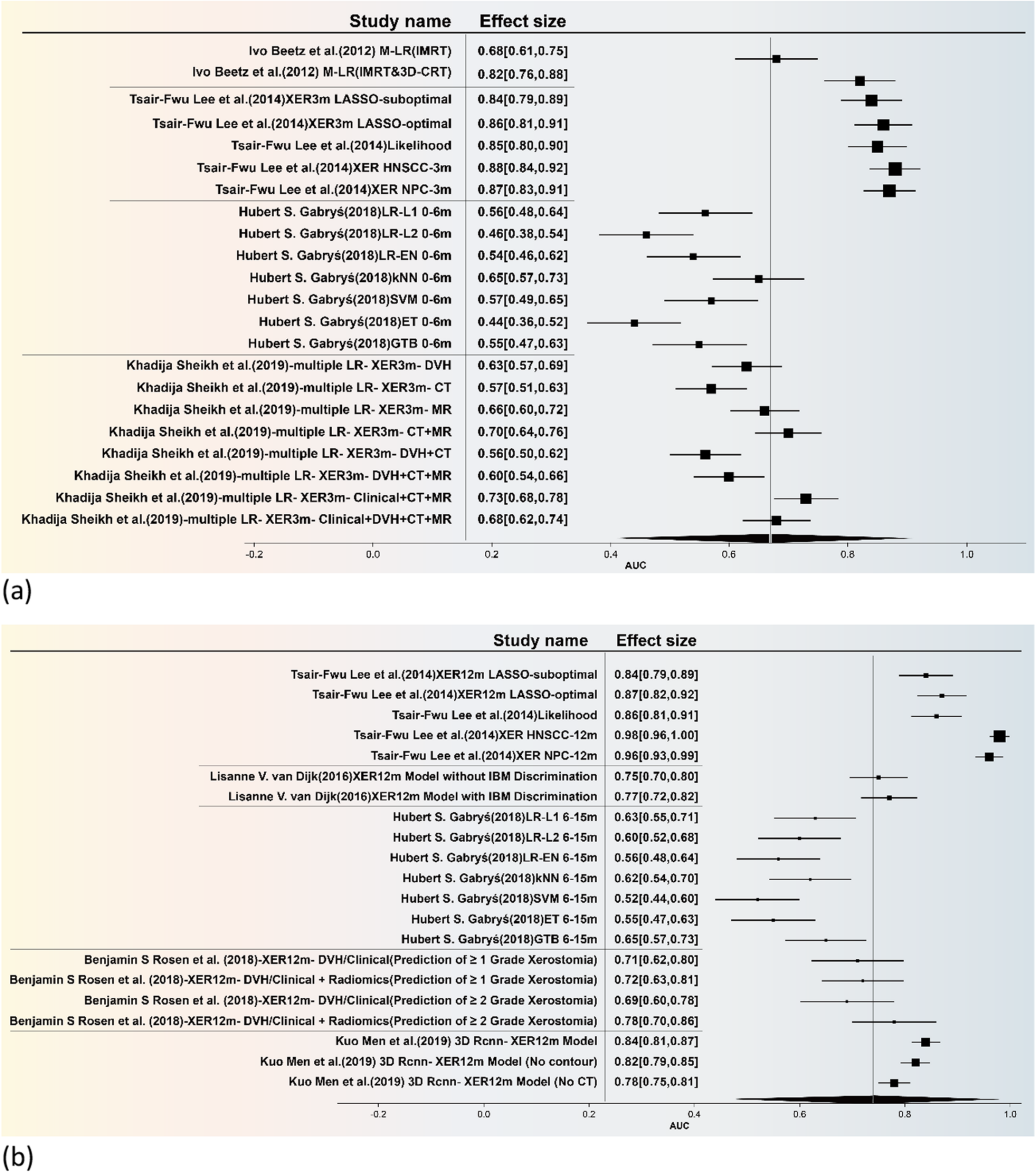
Forest plot **a** the overall effect size for the Area Under the Curve (AUC) of early-effect xerostomia models **b** For late-effect xerostomia models

The forest plot is illustrated in Fig. 4, the present study undertakes a comprehensive and rigorous meta-analysis, focusing specifically on predictive models for xerostomia. Utilizing a feature table, we meticulously integrated the models employed across various studies and further stratified them into early and late phases for sub-group analysis. The combined effect sizes for these sub-groups are visually represented through forest plots (The funnel plot is included in Additional file 1: Figure S2). The temporal demarcation for these phases was set at six months, based on the seminal work of Hubert S. Gabryś [16].

Statistically speaking, the overall effect size for the Area Under the Curve (AUC) of early-effect xerostomia models (Fig. 4a) was 0.67, with a 95% Confidence Interval (CI) ranging from 0.40 to 0.91. This indicates that these models possess moderate predictive accuracy for early-effect xerostomia. However, the high heterogeneity, as evidenced by an I^2^ value of 80.32% and a Q-statistic of 5.34, suggests significant variability across different studies. For late-effect xerostomia (Fig. 4b), the overall AUC effect size was 0.74, with a 95% CI of 0.46 to 0.98. This result further corroborates the models' relatively high predictive efficacy for late-effect xerostomia. Nevertheless, the exceedingly high heterogeneity (I^2^ = 97.99%, Q-statistic = 52.48) implies that the applicability of these models may be limited across different research settings or patient populations.

In Table 4, titled "Prediction model Risk of Bias in Included Studies," the output for each question represents distinct focal points of work, encompassing a comprehensive evaluation of all critical stages in the development and application of prediction models as assessed by PROBAST. The assessment content is divided into four domains: 1. Participants, 2. Predictive Factors, 3. Outcomes, and 4. Analysis. These domains are further categorized based on three assessment outcomes, primarily labeled as "High Risk," "Low Risk," and "Unclear or Ambiguous."

**Table 4:** Prediction model Risk of Bias in included studies

Author, Year	Risk of Bias				Applicability			Overall	
	1. Participants	2. Predictors	3. Outcome	4. Analysis	1. Participants	2. Predictors	3. Outcome	Risk of Bias	Applicability
Hubert S. Gabrys et al. [16]	+	+	+	** − **	+	+	+	** − **	+
Tsair-Fwu Lee et al., [17]	+	+	+	+	+	+	+	+	+
Tsair-Fwu Lee et al., [18]	+	+	+	+	+	+	+	+	+
Lisanne V. van Dijk et al., [19]	+	+	+	** − **	+	+	+	** − **	+
Stefano Ursino et al., [20]	+	+	+	** − **	+	+	+	** − **	+
Jamie A. Dean et al., [21]	+	+	?	?	+	+	?	?	?
Jamie A. Dean et al., [22]	+	+	** − **	** − **	+	+	** − **	** − **	** − **
Ivo Beetz eta al., [23]	+	+	?	+	+	+	?	?	?
Ivo Beetz eta al., [24]	+	+	** − **	+	+	+	** − **	** − **	** − **
Khadija Sheikh et al., [27]	+	+	+	+	+	+	+	+	+
Ben jamin S. Rosen et al., 2018	+	+	+	+	+	+	+	+	+
Kuo Men et al., [25]	+	+	+	** − **	+	+	+	** − **	+

^*^High risk is denoted by "-"; *Low risk is denoted by " + "; *Unclear or ambiguous is denoted by "?"

Although the overall assessment reveals that only four studies exhibited low risk of bias in their data, with the remainder falling under high risk or unclear categories, it is noteworthy that in terms of applicability, only two included studies were assessed as having a higher risk, while two were categorized as unclear or ambiguous. This suggests that while there may be a pervasive issue of data bias, the applicability of these studies is less frequently compromised, thereby indicating a need for more rigorous methodological scrutiny to enhance the reliability and utility of future prediction models.

## Discussion

### Results of the MA study

In our study, we conducted a comprehensive retrospective analysis to evaluate AI-based predictive models for forecasting post-radiation complications like xerostomia in head and neck cancer patients. Our data revealed significant effect sizes of 0.67 and 0.74 for early and late-stage xerostomia, respectively, with *p*-values below 0.05, highlighting the distinctiveness of AI-based models in this context.

Interestingly, our findings contrast with earlier research by our team (Lee et al. [17, 18]) and Van Dijk et al. [19] We observed that incorporating image biomarkers, such as pre-processed CT data, did not necessarily enhance predictive accuracy compared to models solely based on traditional clinical factors and machine learning algorithms. This discrepancy may stem from variations in dataset composition and algorithmic parameters during model training and validation.

Further, research by Gabry et al. [16] identified key features like dosimetric shapes and salivary gland volume through algorithmic comparisons, reiterating the significant divergence between AI-based and traditional clinical models in xerostomia prediction.

However, our study also revealed certain limitations and challenges. Firstly, the limited scope of databases for literature search led to incomplete data and insufficient literature, restricting our ability to perform comprehensive meta-analyses and forest plot illustrations. Secondly, some studies lacked complete data, such as predictive confidence intervals, which further impacted our analysis. Just as per any other site, CNS NTCP literature suffers the same limitations, and no AI has been successfully implemented as yet [28]. Overall, while our study made progress in predicting normal tissue complications after radiotherapy for head and neck cancer, further research and validation are needed. Our findings align with Chulmin Bang's 2023 literature review, emphasizing that the clinical application of AI models still requires more in-depth exploration and validation [29].

### Performance of the CNN-NLP model, optimizer optimization, and coverage

In this study, we presented an analysis focusing on the coverage rate of imbalanced datasets. Despite optimizing the algorithmic parameters, we abstained from employing data augmentation techniques like oversampling or undersampling to bolster the model's predictive accuracy. Our text classification model was conceptualized based on the research framework proposed by Yujia Bao, MA [3]. It's worth noting that this CNN-based model predominantly relies on abstracts rather than full texts for analysis. Consequently, the conversion rate of the included literature could be susceptible to variations in research themes and inclusion criteria, a limitation also acknowledged in Yujia Bao's work [3]. Nevertheless, recent advancements in large-scale language models such as GPT-3 and GPT-4 have shown capabilities in recognizing diverse file formats, including PDFs [30], and have exhibited remarkable precision in medical text identification [30, 31]. Progress has also been made in the realm of deep learning for medical text analysis, exemplified by CNN-based medical report retrieval studies [32]. These technological strides open new avenues for medical text identification, potentially mitigating the aforementioned limitations. We are currently exploring the development of models designed for automated full-text reviews to further enhance the comprehensiveness and accuracy of literature analyses.

## Conclusion

In this study, we employ an integrative approach combining MA and NLP to explore feature factors for NTCP in head and neck cancer. Our results reject the null hypothesis ${H}_{0}$, confirming that specific model-feature combinations yield high predictive accuracy for identical complications. Utilizing CNNs in NLP, we streamline the meta-analytical process and introduce a time-based metric, words per minute (WPM) [33], for efficiency evaluation. This study underscores the utility of meta-analysis and NLP in clinical research, offering a methodological advancement for future studies aiming to optimize predictive models and operational efficiency.

### Supplementary Information

**Additional file 1 Table S1. **Database Retrieval Detail Sheet. **Table S2**. Optimizer Test Set Performance Comparison Table. **Table S3**. Screening speed measured in Words Per Minute (WPM). **Figure S1**. Code for WPM Calculation Algorithm captured from the monitor. Supplementary **Figure S2**. Bias funnel chart for the **a** early -effect **b** late-effect xerostomia

## Data Availability

Not applicable.
