# Supplementary material for: Using meta-analysis and CNN-NLP to review and classify the medical literature for normal tissue complication probability in head and neck cancer
Source: Radiat Oncol. 2024 Jan 9;19:5. doi: 10.1186/s13014-023-02381-7 (PMC10775485; doi:10.1186/s13014-023-02381-7)
Supplement: Supplementary file 1 — Additional file 1 Table S1. Database Retrieval Detail Sheet. Table S2. Optimizer Test Set Performance Comparison Table. Table S3. Screening speed measured in Words Per Minute (WPM). Figure S1. Code for WPM Calculation Algorithm captured from the monitor. Supplementary Figure S2. Bias funnel chart for the a early -effect b late-effect xerostomia [file 13014_2023_2381_MOESM1_ESM.docx]

Supplementary

Supplementary Table S1. Database Retrieval Detail Sheet


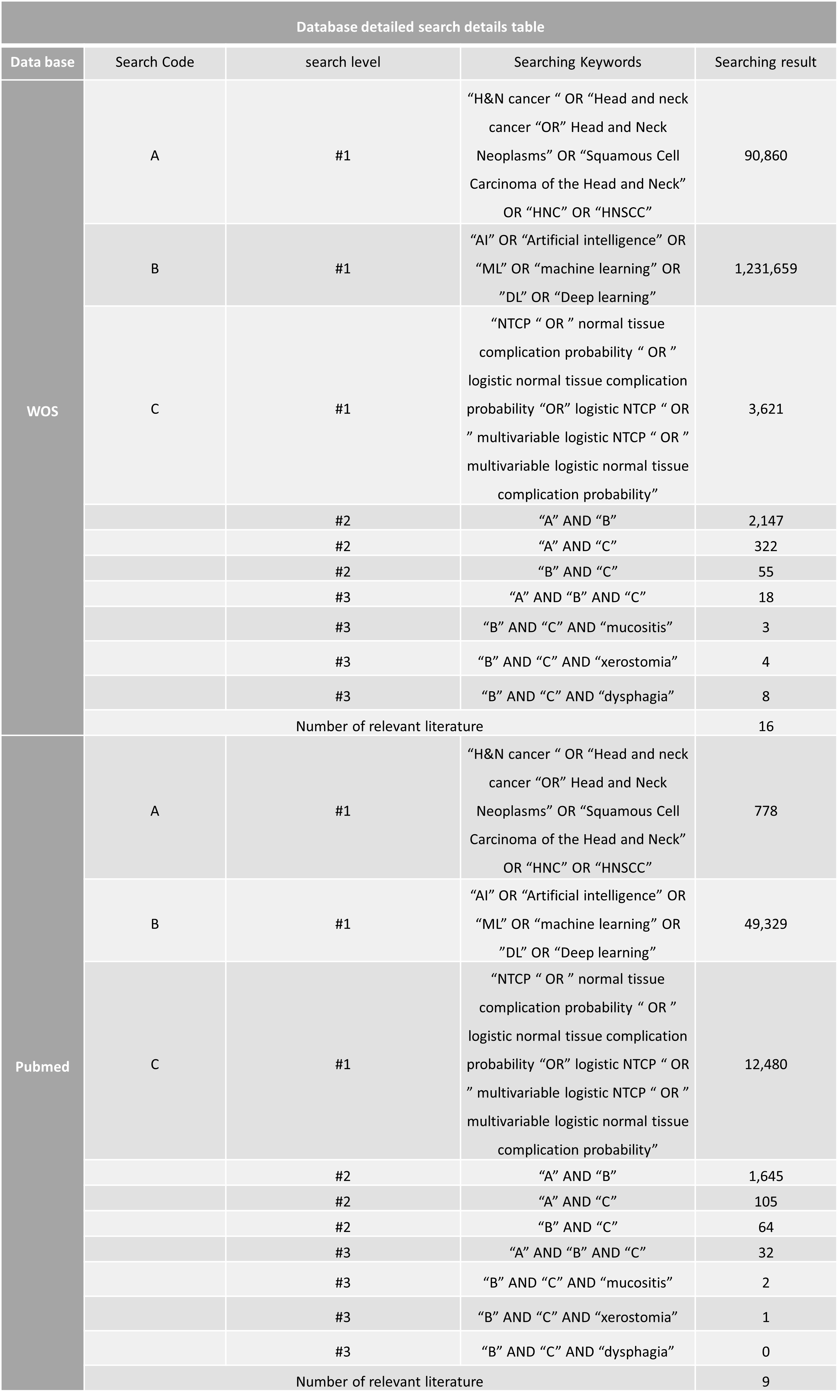


| **Supplementary Table S2**. **Optimizer Test Set Performance Comparison Table** | | | | | | | |
| --- | --- | --- | --- | --- | --- | --- | --- |
| optimizer | epoch | Loss | precision | F1 | ACC | Recall | time used(sec) |
| Adam | 50 | 0.575 | 0.831 | 0.659 | 0.615 | 0.615 | 85.02 |
|  |  | 0.834 | 0.897 | 0.897 | 0.897 | 0.897 | 85.11 |
|  | 100 | 0.569 | 0.836 | 0.682 | 0.641 | 0.641 | 80.36 |
|  |  | 0.643 | 0.930 | 0.914 | 0.923 | 0.923 | 166.97 |
|  | 200 | 0.561 | 0.841 | 0.705 | 0.667 | 0.667 | 358.94 |
| Adadelta | 50 | 1.385 | 0.032 | 0.055 | 0.180 | 0.180 | 86.15 |
|  |  | 1.410 | 0.032 | 0.055 | 0.180 | 0.180 | 85.91 |
|  | 100 | 1.354 | 0.032 | 0.055 | 0.180 | 0.180 | 169.4 |
|  |  | 1.407 | 0.032 | 0.055 | 0.180 | 0.180 | 168.03 |
|  | 200 | 1.400 | 0.032 | 0.055 | 0.180 | 0.180 | 333.88 |
| SGD | 50 | 1.314 | 0.032 | 0.055 | 0.180 | 0.180 | 102.4 |
|  |  | 1.402 | 0.032 | 0.055 | 0.180 | 0.180 | 83 |
|  | 100 | 1.235 | 0.032 | 0.055 | 0.180 | 0.180 | 168.25 |
|  |  | 1.392 | 0.032 | 0.055 | 0.180 | 0.180 | 160.17 |
|  | 200 | 1.371 | 0.032 | 0.055 | 0.180 | 0.180 | 335.9 |
| RMSprop | 50 | 0.982 | 0.032 | 0.055 | 0.180 | 0.180 | 52.2 |
|  |  | 0.619 | 0.836 | 0.682 | 0.641 | 0.641 | 82.53 |
|  | 100 | 0.774 | 0.860 | 0.359 | 0.359 | 0.359 | 47.33 |
|  |  | 0.514 | 0.887 | 0.728 | 0.692 | 0.692 | 162.31 |
|  | 200 | 0.532 | 0.836 | 0.682 | 0.641 | 0.641 | 253.26 |
| Adagrad | 50 | 0.627 | 0.852 | 0.750 | 0.718 | 0.718 | 85.12 |
|  |  | 1.183 | 0.032 | 0.055 | 0.180 | 0.180 | 84.31 |
|  | 100 | 0.557 | 0.841 | 0.705 | 0.667 | 0.667 | 166.63 |
|  |  | 1.106 | 0.032 | 0.055 | 0.180 | 0.180 | 166.5 |
|  | 200 | 1.011 | 0.032 | 0.055 | 0.180 | 0.180 | 322.35 |
| AdamW | 50 | 0.636 | 0.889 | 0.841 | 0.872 | 0.872 | 164.52 |
|  |  | 0.825 | 0.870 | 0.795 | 0.846 | 0.846 | 83.79 |
|  | 100 | 0.636 | 0.889 | 0.841 | 0.872 | 0.872 | 164.52 |
|  |  | 0.641 | 0.909 | 0.880 | 0.897 | 0.897 | 173.4 |
|  | 200 | 0.574 | 0.831 | 0.659 | 0.615 | 0.615 | 332.01 |
| Adamax | **50** | **0.510** | **0.852** | **0.750** | **0.718** | **0.718** | **86.06** |
|  |  | **0.964** | **0.032** | **0.055** | **0.180** | **0.180** | **86.46** |
|  | **100** | **0.603** | **0.865** | **0.794** | **0.769** | **0.769** | **86.45** |
|  |  | **0.745** | **0.870** | **0.795** | **0.846** | **0.846** | **168.85** |
|  | **200** | **0.629** | **0.952** | **0.945** | **0.949** | **0.949** | **344.53** |
| ASGD | 50 | 1.313 | 0.032 | 0.055 | 0.180 | 0.180 | 86.23 |
|  |  | 1.402 | 0.032 | 0.055 | 0.180 | 0.180 | 86.61 |
|  | 100 | 1.235 | 0.032 | 0.055 | 0.180 | 0.180 | 169.94 |
|  |  | 1.392 | 0.032 | 0.055 | 0.180 | 0.180 | 169.31 |
|  | 200 | 1.371 | 0.032 | 0.055 | 0.180 | 0.180 | 337.11 |
| Rprop | 50 | 0.640 | 0.831 | 0.659 | 0.615 | 0.615 | 62 |
|  |  | 0.639 | 0.841 | 0.705 | 0.667 | 0.667 | 77.06 |
|  | 100 | 0.685 | 0.831 | 0.659 | 0.615 | 0.615 | 57.03 |
|  |  | 0.615 | 0.836 | 0.682 | 0.641 | 0.641 | 81.75 |
|  | 200 | 0.629 | 0.835 | 0.790 | 0.769 | 0.769 | 76.86 |

| **Supplementary Table S3.** **Screening speed measured in Words Per Minute (WPM)** | | |
| --- | --- | --- |
| **author** | **Study title** | **Wpm**  **( Words per minute )** |
| Hubert S. Gabryś et al. | Design and Selection of Machine Learning Methods Using Radiomics and Dosiomics for Normal Tissue Complication Probability Modeling of Xerostomia | 112.08 |
| Tsair-Fwu Lee et al. | LASSO NTCP predictors for the incidence of xerostomia in patients with head and neck squamous cell carcinoma and nasopharyngeal carcinoma | 91.41 |
| Tsair-Fwu Lee et al. | Using Multivariate Regression Model with Least Absolute Shrinkage and Selection Operator (LASSO) to Predict the Incidence of Xerostomia after Intensity-Modulated Radiotherapy for Head and Neck Cancer | 123.97 |
| Lisanne V. van Dijk et al. | CT image biomarkers to improve patient-specific prediction of radiation induced xerostomia and sticky saliva | 100.42 |
| Stefano Ursino et al. | Incorporating dose–volume histogram parameters of swallowing organs at risk in a video fluoroscopy-based predictive model of radiation-induced dysphagia after head and neck cancer intensity-modulated radiation therapy | 113.59 |
| Jamie A. Dean et al. | Incorporating spatial dose metrics in machine learning-based normal tissue complication probability (NTCP) models of severe acute dysphagia resulting from head and neck radiotherapy | 100.84 |
| Jamie A. Dean et al. | Normal tissue complication probability (NTCP) modelling using spatial dose metrics and machine learning methods for severe acute oral mucositis resulting from head and neck radiotherapy | 128.60 |
| Ivo Beetz et al. | NTCP models for patient-rated xerostomia and sticky saliva after treatment with intensity modulated radiotherapy for head and neck cancer: The role of dosimetric and clinical factors | 114.23 |
| Ivo Beetz et al. | Development of NTCP models for head and neck cancer patients treated with three-dimensional conformal radiotherapy for xerostomia and sticky saliva: The role of dosimetric and clinical factors | 120.81 |
| Kuo Men et al. | A Deep Learning Model for Predicting Xerostomia Due to Radiation Therapy for Head and Neck Squamous Cell Carcinoma in the RTOG 0522 Clinical Trial | 100.46 |
| Benjamin S Rosen et al. | Early Changes in Serial CBCT-Measured Parotid Gland Biomarkers Predict Chronic Xerostomia After Head and Neck Radiation Therapy | 115.21 |
| Khadija Sheikh et al. | Predicting acute radiation induced xerostomia in head and neck Cancer using MR and CT Radiomics of parotid and submandibular glands | 99.94 |
| Average wpm | | 110.13 |


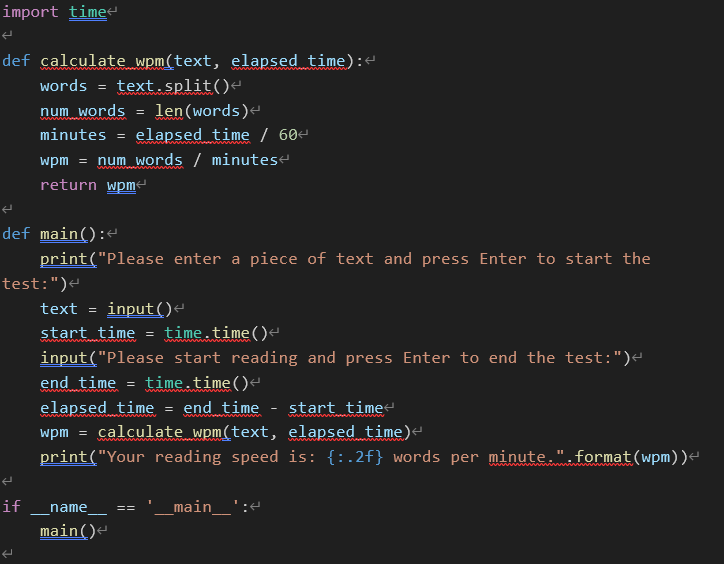


**Supplementary Figure S1. Code for WPM Calculation Algorithm captured from the monitor**


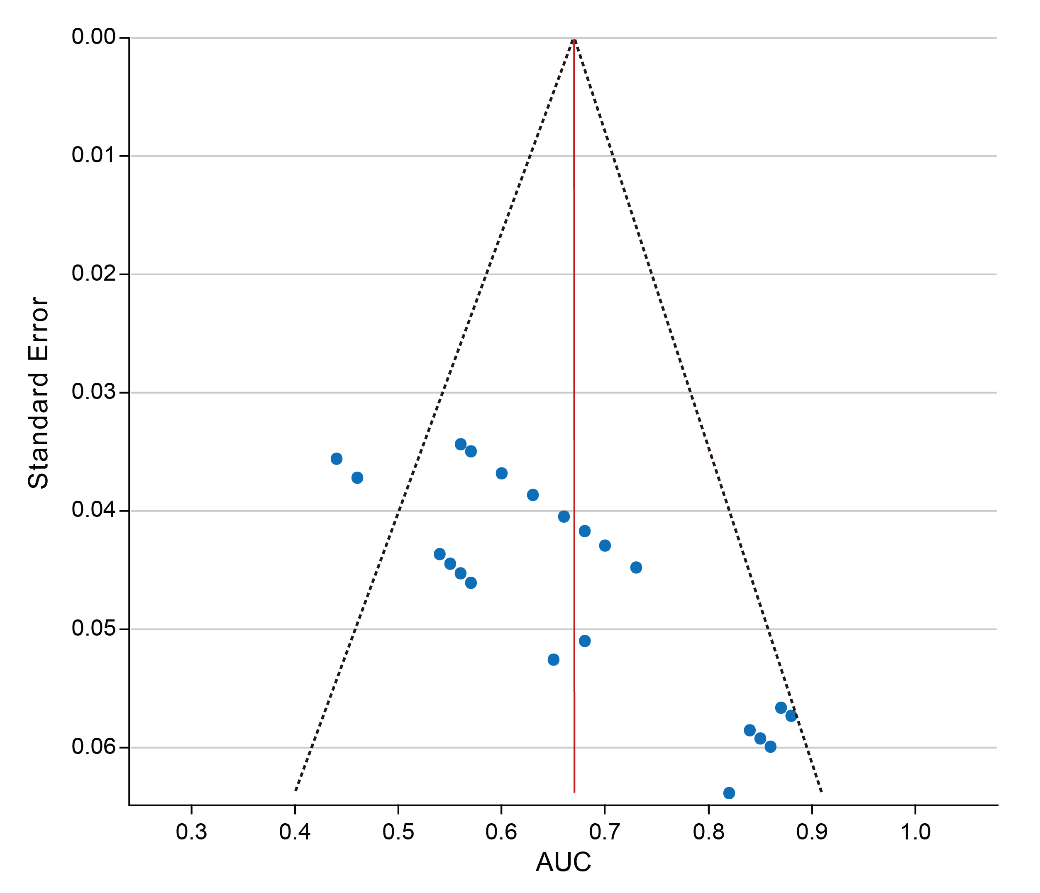


(a)


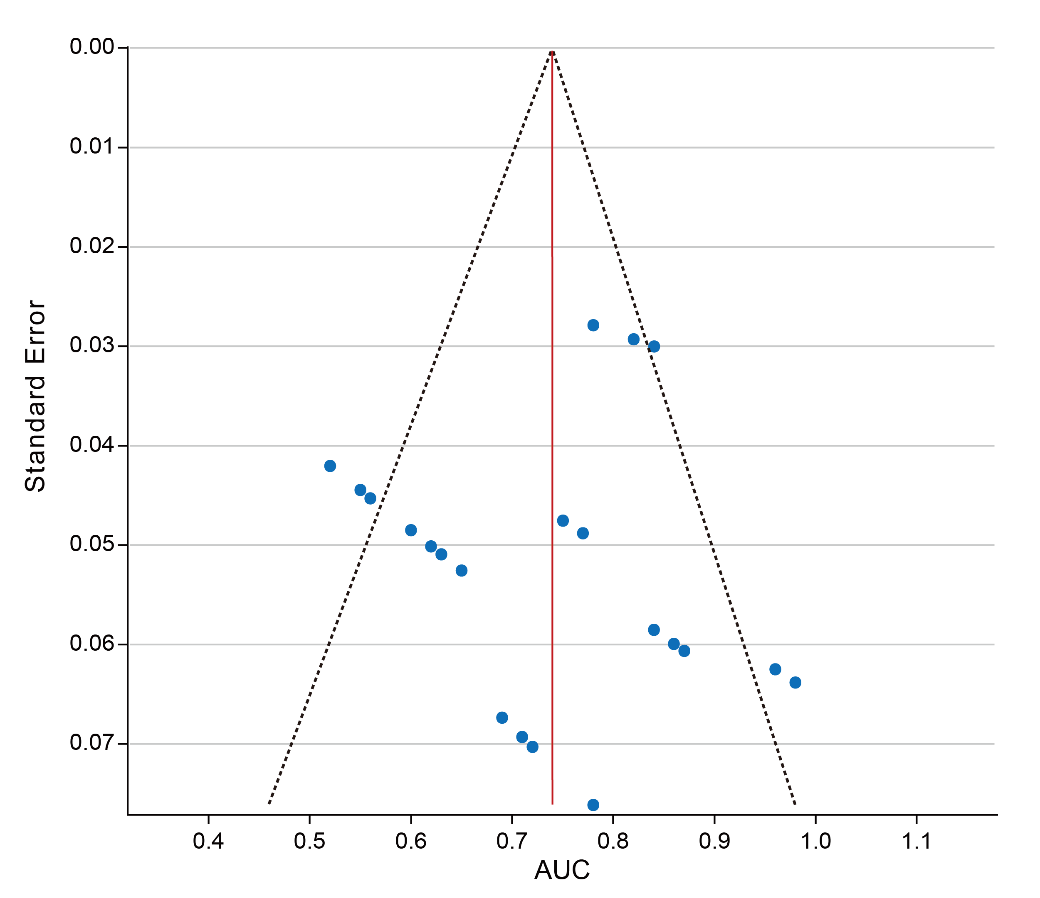


(b)

**Supplementary Figure S2. Bias funnel chart for the (a) early -effect (b) late-effect xerostomia**
